# Review of Emergency Medical Services Vulnerability to High Consequence Infectious Disease in the United States

**DOI:** 10.3389/fpubh.2021.748373

**Published:** 2021-10-05

**Authors:** Thomas W. Richey, Raymond L. Fowler, Ray E. Swienton, James Patrick O'Neal, Curtis A. Harris

**Affiliations:** ^1^Institute for Disaster Management, College of Public Health, University of Georgia, Athens, GA, United States; ^2^Emergency Medicine Center at Dallas, University of Texas Southwestern Medical Center, Dallas, TX, United States

**Keywords:** emergency medical service (EMS), high consequence infectious diseases (HCID), standard precaution among EMS, EMS vulnerability, EMS licensure and education

## Abstract

**Purpose:** Emergency medical services (EMS) responders are a group of medically skilled professionals who perform a wide range of essential medical services within a community including emergency response, patient transport, and mobile integrated healthcare. The proper functioning of the EMS system is paramount to the well-being of the medical system and public health. The intent of this paper is to review current EMS standards and practice to determine the danger a high consequence infectious disease (HCID) may pose to these healthcare workers and the community.

**Areas Addressed:** Through the review of EMS practice several areas were identified as vulnerabilities to the EMS network. These vulnerabilities consisted of the lack of standardized licensing practice, inconsistent medical direction, and the inability to properly implement the use of personal protective equipment (PPE). The compounding of these vulnerabilities allows for HCIDs to pose a serious threat to EMS personnel with the possibility of devastating and crippling the EMS infrastructure within the US.

**Discussion:** The vulnerabilities identified must be addressed both to protect EMS providers and to enhance the resilience of the US healthcare system. Ways to address the identified vulnerabilities should focus on improving the EMS curriculum and increasing minimum levels of education for first responders. Targeting minimum education and training standards could be the most effect method of reducing the dangers of HCIDs to EMS systems.

## Introduction

Emergency medical services (EMS) responders are medically skilled professionals who respond to the scenes of disasters and other emergencies to provide assistance and medical care. These professionals are not limited to emergency response work strictly and provide necessary transportation services for patients from the prehospital setting to a hospital, from one hospital to another, or from a hospital to other institutions such as long-term care facilities. Additionally, many EMS personnel operate mobile integrated healthcare operations as a function of community paramedicine efforts. Given the critical role responders play in the medical transport structure, the disruption of EMS within a given area would be catastrophic to the functioning of the industrial-medical complex, and the communities it serves. With this consideration, it is essential to highlight threats to the continuity of the EMS infrastructure (1, 2).

Individual EMS responders face a wide array of hazards when responding to an emergency or disaster. However, of the hazards faced, few may pose as significant a threat systematically as high consequence infectious diseases (HCIDs). According to Brouqui (3), “highly infectious diseases are transmissible from person to person, cause life-threatening illness and present a serious hazard in healthcare settings and the community, requiring specific control measures.” HCIDs have the potential to cripple an EMS system's ability to respond to emergencies and transport patients effectively. One such example of the devastating effects of HCIDs involves the Severe Acute Respiratory Syndrome (SARS) outbreak in Toronto, Canada, in 2003. It was estimated that during the outbreak, roughly 850 paramedics experienced ~1,166 SARS exposures. These exposures resulted in the 10-day quarantine of 436 EMS providers, effectively cutting the staffing and limiting the ability of Toronto EMS to effectively respond to the epidemic. In the cohort of 436 paramedics that were quarantined, some 60 paramedics developed SARS (4). The SARS event in Canada demonstrated that HCIDs, even those with lower rates of transmissibility, pose a significant danger to the public health and can necessitate the quarantine of a large portion of EMS staff, substantially affecting the ability of the EMS system to respond to the public it serves.

The ability of HCIDs to cripple functioning society is not a new experience. HCIDs have tormented humankind since the dawn of civilization. Events such as the Black Death of the Middle Ages, the Spanish Flu of 1918, and smallpox in the New World have drastically altered human history (5, 6). In response, considerable efforts have been placed on advancing medical technology and improving healthcare providers' success rates in treating HCIDs. Unfortunately, the rate of novel and reemerging HCIDs has only increased, while factors such as global trade, transit, and urbanization have notably aided the propensity of HCIDs to spread (6).

EMS providers serve as the rapid response arm of the healthcare system. If an HCID outbreak were to occur within a community, EMS providers would often be the first to come into contact with patients and could provide forewarning to the rest of the medical community (7). Unfortunately, the current United States (US) EMS network system suffers from notable issues that may prevent EMS providers from safely, quickly, and accurately addressing HCID events. Some areas of concern that hinder EMS responders include their authorized scope of practice, educational and training standards, and public health awareness related to HCIDs. The inadequate implementation of standard precautions by EMS providers is also of great concern. For example, difficulty maintaining appropriate precautions against disease transmission may be compounded in an HCID outbreak, during which EMS providers might be required to increase their level of personal protection to address specific contact, droplet, and airborne precautions (7).

The ongoing COVID-19 pandemic demonstrates the capability of an HCID to stress and challenge the EMS network and its personnel (8). This pandemic has demonstrated the importance of EMS systems in the containment of an HCID through the use of phone triage, home testing, drive-in testing, and reorganization of the EMS structure (9, 10). Given EMS providers' necessary presence during HCID outbreak response, it is of paramount concern to assess EMS vulnerabilities and develop plans for future improvement.

## Review

### EMS Licensing and Educational Requirements

The Highway Safety Act of 1970 expanded the Department of Transportation (DOT) to include the National Highway Traffic Safety Administration (NHTSA). The NHTSA was established to aid at the federal level in the development of EMS systems. To address the need for EMS reform, NHTSA primarily focused on establishing recommendations for the education and training of prehospital providers. These recommendations laid the foundation for establishing modern EMS practice in America (1, 2). The development of standardized EMS education and training curricula eventually led to the 2005 National EMS Scope of Practice Model. The model proposed four different levels of training for Emergency Responders. Each level would require advancing education and training standards, ultimately resulting in varying scopes of practice, which were defined by certificate or license the states issued a provider.

The four basic training levels proposed, from the most restricted to the broadest scope of practice, are as follows: Emergency Medical Responder (EMR), Emergency Medical Technician (EMT), Advanced Emergency Medical Technician (AEMT), and Paramedic. The goal of establishing these levels was to allow EMS agencies to better match a provider's scope of practice to the level of care needed during an emergency response (1, 2). While NHTSA has produced recommendations for education and training requirements, it ultimately falls to the states to certify or license providers and determine the approved educational standards and scope of practice for specific levels. Due to a lack of uniform licensing standards for EMS providers across the country, variability exists within EMS networks across the country for providing services during a large-scale disaster (11).

Further compounding the issues the US currently faces during the COVID-19 pandemic is the meager amount of HCID education in the current federal recommendations. Currently, only 1.3% of the material covered in the NHTSA education standards are related to training on the varying nature of infectious diseases. This amount of education on HCIDs may be insufficient for EMS responders who must evaluate and manage HCID outbreaks (11). For instance, EMS providers arriving on the scene generally have not received a medical history or diagnosis for the patient needing care (12). The lack of prior medical history and diagnosis creates an environment that demands a responder be able to quickly and accurately obtain a patient's medical, travel, and relevant exposure history to determine the risk the patient may pose to others. The ability to complete such vital assessments is hampered due to both time constraints and the amount of training in HCIDs. EMS responders may incidentally become victims of disease outbreaks due to the difficulty of accurately identifying HCIDs at the scene of emergencies. On a call, responders often face numerous responsibilities, such as dealing with critical patients while working in potentially unsafe work environments. It is essential for the providers' well-being that they maintain awareness that any patient may have an HCID (11).

EMS systems throughout the US vary significantly in structure and organization. Five broad types of EMS agencies are generally recognized: fire-based services, government-based services (e.g., public utility models and municipal systems), privately owned services, volunteer services, and hospital-based services (12), with variability across regions based upon the evolution of systems across populations. In the US, roughly 40% of EMS is provided by fire-based agencies, and at least one-third of states rely on volunteer EMS agencies to varying degrees (13). The variations that exist among how EMS services are provided may create difficulties in the prompt dissemination of up-to-date educational and training materials. The lack of a prompt ability to provide timely updates related to HCIDs may place EMS responders—and the communities they serve—at an increased risk of exposure (11).

Variability among modern EMS systems may be exacerbated by inconsistencies with EMS agencies' medical direction. EMS medical directors are responsible for establishing practices and training requirements for EMS agencies. Inconsistent requirements for EMS medical directors may foster variability in the levels of care provided by EMS agencies. For example, some states have specific requirements for EMS systems' medical directors, such as board certification in emergency medicine and/or EMS medicine. In contrast, other states only require that the medical director be a licensed physician, a condition often prompted by the lack of physicians willing to provide EMS medical direction. This variability in EMS medical director training and background may contribute to variations in EMS systems' capabilities across regions (14, 15). For example, during large scale disasters, EMS agencies play a critical role in stabilizing healthcare infrastructure in disaster-affected areas. An effective response to such large scale events often requires several EMS agencies to work in joint operations, which cross pre-existing regional EMS jurisdictions. Historically, integrating these diverse EMS resources has been limited by non-interoperable protocols and guidelines (16). The interplay between EMS jurisdictions necessitates an over-arching medical direction and scope of practice to enable EMS agencies to work cohesively together. While NHTSA has established a national scope of practice for EMS providers, a concerted effort is needed to coordinate medical direction standards and practices (17).

EMS providers' ability to take an active role in preventing or mitigating an HCID outbreak is affected in part by the amount of time spent receiving education about HCIDs, the ability to distribute new and updated information related to HCIDs, and specific focus toward the management of HCID outbreaks. When EMS providers are actively engaged in detecting and identifying HCIDs that may arise in communities, hospitals can be appropriately notified by inbound EMS units, to allow preparation for the isolation of patients upon arrival. As outlined in the EMS Infectious Disease Playbook, inbound EMS providers should alert hospitals well-prior to completing the transport of possible HCID cases (7). This notification process should also extend from hospitals to EMS providers. Hospitals must be accountable to EMS agencies; if a patient previously transported is subsequently diagnosed with an HCID, hospitals should be required to notify the transporting EMS agency. This notification would allow EMS agencies to protect providers by providing targeted prophylaxis and quarantining if indicated. The ability of hospitals and EMS agencies to exercise bi-directional communication concerning the presence of HCIDs in the community is necessary to increase the medical community's resilience while also protecting both EMS and hospital employees.

### Standard Precautions for EMS Responders

As previously mentioned, EMS responders are often unaware of the communicable diseases which patients could be harboring (12). The lack of prehospital awareness of the potential for communicable diseases in a community necessitates that EMS providers strictly adhere to the use of standard precautions (18). Standard precautions include a set of strict actions and personal protective equipment (PPE) that healthcare providers implement to reduce exposure risk (7).

Standard precautions may be summarized as hand hygiene, proper PPE use, awareness of the potential for aerosols being exhaled by patients, sharps safety, and proper cleaning and disinfecting of environmental surfaces. The use of proper standard precautions offers protection to both EMS responders and patients being encountered. Correctly implementing all aspects of standard precautions can help break the cycle of infection while reducing the risk of spreading infection during patient care (19).

Although hand hygiene is one of the most critical components of infectious disease control, proper handwashing remains an issue that requires more focus within the medical community. Throughout their days, EMS providers regularly enter both hospital and community settings. This fluctuating environment creates the risk of cross-contamination within both the hospital and the community. EMS responders unable to employ proper hand hygiene could quickly spread a community-acquired infection into a hospital or transmit nosocomial infections (those acquired in a hospital) into a community (20). EMS providers, like many healthcare providers, have been noted to have less than optimal hand hygiene. In a study in 2015 polling EMS providers about personal hand hygiene practices, around 13% of EMS providers stated they regularly washed their hands prior to patient care, 54% washed hands after skin contact with a patient, 67% washed hands after completing a call, and only 33% cleaned their hands after an invasive procedure (20). While this study was a survey, nevertheless, these results are troubling because those responses showed sub-standard hand hygiene practices by the participants of that survey, which shows a risk that could expose the providers, the communities, and their families to a higher likelihood of infection.

PPE is included as part of standard precautions. PPE can include gloves, gowns, and mouth, nose, and eye protection. PPE functions to create a barrier between the patient and the provider to prevent the spread of disease. While PPE can be effective at preventing the spread of infectious disease, it requires providers to correctly identify the level of PPE needed and correctly implement PPE use. If incorrect PPE is selected or improperly implemented, the PPE may fail to prevent infectious disease spread. Working in a time-critical setting—such as the prehospital environment—makes it challenging for responders to implement PPE use properly. According to Harris and Nicolai (21), common reasons for improper PPE use stated by EMS providers included “time required for correct use, impairment of vision or movement, and forgetfulness.” Gloves are viewed as the most essential and basic PPE that should be used by healthcare providers. However, in two separate studies, correct glove use was documented only 52 and 56% of the time (20, 22). These low levels of compliance for proper glove use pose a direct threat to both providers and patients' well-being. Critically crucial, along with the selection of PPE, is the capability of implementing the PPE. The implementation of PPE is completed through the process of “donning” and “doffing.” Donning and doffing refer to the critical task of correctly putting on (donning) and taking off (doffing) PPE. The proper donning and doffing of PPE is necessary to prevent HCIDs from contaminating EMS providers. If a critical error were to occur during donning or doffing, providers could easily become contaminated, which effectively nullifies the purpose of PPE. In a previous study, EMS providers were tested on their ability to don and doff PPE in a practice exercise correctly. Test participants performed poorly, with only 14.3% of participating EMS providers able to correctly don and doff PPE without committing a critical error (23). These results underscore a need for significant improvement in PPE education and training for EMS responders. HCIDs also pose a serious concern related to the need for specialized PPE that many EMS agencies may struggle to access. For an HCID, such as Ebola Virus Disease (EVD), the CDC issued unique PPE recommendations related to the uses of impermeable gowns and/or coveralls, surgical hoods, and other PPE that may not be regularly stocked by EMS agencies (24). The combination of underutilized PPE, the need for specialized PPE, and the inability to safely implement PPE display the vulnerabilities currently within EMS practice.

The careful management of sharps directly relates to EMS providers' protection from the dangers of bloodborne pathogens. The Centers for Disease Control and Prevention (CDC) and the National Institute for Occupational Safety and Health (NIOSH) have identified needle stick injuries (NSIs) as a significant concern for healthcare workers. In the US, an estimated 600,000 to 800,000 needlestick injuries occur annually within the healthcare workforce, with as many as 50% of cases being unreported (21). EMS responders attempting to perform delicate medical procedures often in a mobile environment are at an increased risk for NSIs. In 1991 OSHA established that contaminated needles should not be recapped; following this policy, both NIOSH and CDC recommended providers avoid recapping needles after use to prevent NSI events (19, 25, 26). During one study, ~20% of EMS providers stated that they followed federal recommendations and did not recap needles after use (21). The lack of compliance with the capping policy, along with suspected under-reporting of NSI events by healthcare workers, creates an environment ripe for the spread of bloodborne pathogens and HCID infections.

Standard precautions also require the maintenance of a clean, disinfected environment. Compliance with this part of standard precautions may be difficult for EMS providers caring for the acutely ill and injured in the field. EMS responders engaging in patient care in the prehospital environment are responsible for the cleanliness and disinfection of ambulances and equipment prior to during, and after patient care and transport. If ambulances are improperly sanitized, they may serve as reservoirs for infectious diseases and a breeding ground for multidrug-resistant microbes. Thus, it is necessary that EMS providers adequately disinfect ambulances prior to response and then again after transporting a patient. When examining the bacteria present in ambulances, several studies found some level of contamination. While the bacteria's levels differed within each study, all studies found some form of Staphylococcus present in ambulances. Some studies detected the presence of Klebsiella and *E. coli* (27–30). The presence of these and other pathogenic bacteria in ambulances is a danger to patients' and providers' health. Equally concerning is the significant mobility of bacteria that has been demonstrated in previous research. A pathogen's capability to spread within the ambulance environment was verified in a study that used a bacteriophage tracer to examine the spread of a pathogen during EMS responses. The researchers placed the tracer on two frequently used surfaces in an ambulance. Upon completion of emergency calls, the researchers then evaluated the ambulance for the tracer's presence outside of the previously contaminated sites. Upon evaluating the ambulances, the study found that cross-contamination of ambulance surfaces was evident in 100% of calls (12).

In addition to the cleaning and maintenance of ambulances, EMS providers must also be diligent in the cleaning and disinfecting of equipment used by EMS staff. In one study, researchers found that roughly 32% of stethoscopes used by EMS providers were contaminated with methicillin-resistant *Staphylococcus aureus*. In another study, 32% of EMS providers were unable to document the last time that medical equipment had been cleaned (31). The combination of pre-existing contamination in the EMS environment, the lack of consistent cleaning by EMS staff, and the ability of pathogens to cross-contaminate ambulance surfaces highlight the need for EMS providers to be adequately trained in disinfection and cleaning of ambulances and equipment and to be held accountable for doing so under well-established and monitored agency policies.

## Discussion

Emergency Medical Services providers have long been accepted as integral members of the broad medical community. Functioning in the emergency response and transport component of the medical infrastructure, EMS providers are often the first medical providers to interact with emerging illnesses within a community. Emerging community diseases can vary from mundane illness to life-threatening HCIDs. EMS care providers face a wide array of uncontrolled variables related to a patient's medical condition and medical history. These uncertainties contribute to the importance of EMS practice standards in protecting providers from a wide range of risks. By evaluating current EMS practice, two major elements were identified as vulnerabilities in EMS safety and leading to increased risk related to HCIDs. The first practice element identified was the current lack of consistency and depth in EMS licensing standards and educational requirements. This lack of uniformity in the areas of licensing standards and educational requirements has created EMS systems that function at different levels of capability, which presents a significant potential threat during an HCID outbreak scenario. The second area of concern relates to the documented inability of EMS providers to implement standard precautions properly. This failure to maintain standard precautions increases the prospect of an EMS provider contracting an HCID and creating a substantial risk of spreading the infection within the community and elsewhere. The intersection of these failures in licensing standards, educational requirements, and standard precautions compliance exacerbates the danger and increases the hazard HCIDs pose to EMS systems.

Each failure creates a unique type of danger for EMS professionals. For example, the inconsistency of state licensing standards' creates variability across jurisdictional boundaries, which can complicate and hinder an HCID outbreak response. For instance, if a national entity, such as the Centers for Disease Control and Prevention (CDC), established a recommendation for suspected HCID patients' treatment during EMS response, inconsistent levels of licensing and training could decrease adherence to national guidance. The current lack of licensing uniformity creates regional capability variations, leaving some areas unable to meet the national recommendations due to an unequal capability produced by non-uniformed State licensing practices. The risk associated with irregular state licensing practices is compounded by a significant lack of coordinated educational guidance covering HCIDs from the national level. Currently, only 1.3% of NHTSA educational recommendations are related to infectious diseases (11). The current amount of information provided to EMS professionals lacks the necessary depth to convey the dangers that infectious diseases pose to EMS professionals and their communities. Besides education, another method of mitigating risk related to an infectious disease involves using standard precautions to limit the exposure of EMS personnel to infectious disease during patient care and transport; however, the EMS implementation of standard precautions is inconsistent. The low rates of proper standard precaution implementation create a dangerous situation in which an HCID could rapidly spread through the inadequately protected EMS population (20–22). The combination of irregular State licensing, inadequate education requirements, and improper implementation of standard precautions creates serious vulnerabilities to EMS agencies and providers.

Addressing the existing vulnerabilities within the EMS practice must become of paramount concern to medical professionals nationwide. No one solution will completely resolve the current vulnerability gaps that confront EMS agencies across this nation. A possible solution to address the current lack of uniformity affecting State licensing could involve leveraging the current certification format of the National Registry of Emergency Medical Technicians (EMTs) to serve as the basis for all state licensure. Currently, the National Registry of EMTS (NREMT) certification is accepted in all states and serves as the foundation for state licensure in 46 states (32). Using the NREMT certification as the basis for all State's licensure practices could create a common standard and alleviate discrepancies in EMS practice and potentially provide momentum for a nationally recognized EMS license. Creating a more uniform EMS professional license could also be used to address the current lack of education standards related to HCIDs. A method of improving future EMS professionals' educational levels might entail expanding current NHTSA educational guidelines to include topics related to caring for patients with HCIDs and a basic clinical understanding of HCIDs. The expansion of NHTSA educational recommendations could significantly improve EMS providers' future capabilities to safely respond to dangerous HCID outbreaks (11). While modification to EMS educational standards will benefit the future providers in the field, it does little for current active EMS responders.

A possible solution to address the current lack of HCID awareness could be implementing a statewide Infectious Disease Networks (IDN). For example, in 2014, due to the Ebola Virus Disease threat, the State of Georgia created its IDN. Georgia's IDN is a system designed to transport, treat, and evaluate potential HCID patients (33). By delegating these responsibilities, Georgia effectively reduced the risk of HCIDs by limiting the number of contacts to specially trained EMS agencies and hospitals comprising the IDN. Through Georgia's effort, 12 hospitals and 16 EMS agencies had teams of trained providers to administer care to potential HCID patients (33). The establishment and utilization of an IDN appear to be a feasible method for enhancing HCID capabilities to address small-scale or controlled HCID events. However, IDNs may not be successful in improving the resilience of all susceptible EMS services and hospitals within a state. Another option could be to build upon the IDN framework at both the state and federal levels. The federal standards could provide a baseline for each State to prepare for HCID incidents, while still affording autonomy to individualize a state's EMS culture. States might then create distinct, statewide IDNs which would mirror federal policy standards for IDNs. Another solution for improving functional knowledge of HCIDs within the EMS practice could include required HCID training as a necessary part of continuing education credit for all EMS providers. In requiring HCID education as part of relicensing efforts, States could rapidly disseminate educational material to active EMS providers and effectively reduce the risk related to the current lack of educational materials.

In addition to addressing EMS licensing practices and educational requirements, there is a need to improve standard precaution utilization. Currently, the attempt to improve the use of standard precautions is not limited strictly to the EMS profession; instead, the improvement of standard precautions compliance is an ongoing effort in the broader healthcare community (34). Imitating previous efforts made by other healthcare professions is beneficial to effectively guide the development and improvement of EMS uptake of standard precautions. By reviewing standard precaution compliance initiatives, two possible interventions were identified as potential programs to be tailored toward EMS professionals. The two promising interventions are visualization education techniques and peer evaluations; both solutions appear to offer some level of success (34). The first solution utilizes standard education practices in conjunction with the abstract visualization of the spread of disease. In the study of an emergency department staff, it was found that after completing education with the addition of the visualization of the spread of disease, mask use increased to some 74% of encounters rather than the previously documented 53% of encounters (34). Combining the standard education practice with the visualization of disease spread could be especially beneficial for EMS providers due to the drastic spread that can occur in the closed confines in which EMS providers typically work (12).

The second possible intervention could be in utilizing peer evaluations to appraise the EMS staff's use of standard precautions. When implemented in an acute care facility, the establishing of peer evaluation has shown a 33.5% improvement in standard precaution use from the previous baseline at the completion of the intervention and a 24% improvement upon the previous baseline 4 weeks following the completion of the intervention (34). Peer evaluation documentation could enable EMS managers to be better aware of the standard precaution implementation within an agency. These documents would allow directors to provide targeted training and remediation to providers who struggle with implementing standard precautions. It would be hoped that through peer evaluations, EMS agencies can foster an environment focused on the safety and well-being of EMS professionals, with the understanding that through maintaining standard precautions, EMS providers can help prevent the spread of disease. Evidence suggests that increases in EMS provider training related to PPE use lead to an increase in EMS personnel's ability to correctly determine precautionary needs for a response and correctly implement the needed PPE (21). The proper use of PPE and standard precautions when caring for patients in the field can be the difference between a healthy functioning EMS workforce and one incapacitated by an HCID.

The EMS profession is an expansive and diverse vocation playing a significant role in the functioning of the day to day medical-industrial complex in the United States. Due to this profession's size and scope and in consideration of the need for encouraging EMS-specific research, the present study focuses upon specific aspects of the EMS profession as related to HCID vulnerability. This review has been limited to the impact that current state licensing and educational requirements and current standard precaution uses has on HCID vulnerability. Future research should consist of a more in-depth analysis of topics such as the effect agency configuration, which may play a substantial impact upon an agency's capability to address HCID outbreaks. Furthermore, medical oversight is an essential topic influencing this discussion, requiring more in-depth analysis and research to determine the effect medical oversight plays on HCID response broadly. Such research into HCID specific vulnerabilities within the EMS profession will assist in protecting and fortifying EMS systems from the ever-present threat of HCIDs.

Moving forward, it will be of great interest to follow the evolution of the EMS profession. The 2050 EMS Agenda created by the DOT paints an appealing image of the EMS system, stating that “a future EMS System will rely on a strong backbone of responders with training to provide immediate lifesaving care. Supplementing and overseeing that level of response will be a highly educated EMS professional providing more advanced care” (35). The future that is described by the DOT seems to lend itself to the creation—and regular utilization—of baccalaureate trained EMS providers to supplement and oversee the response of EMS systems broadly. The creation and implementation of a standardized baccalaureate-level education for EMS providers poses an exciting possibility for the EMS future. It would be intriguing to examine the effect of standardized baccalaureate-level EMS providers upon the EMS vocation specifically and upon the vulnerability to HCID.

## Conclusion

EMS systems are critically vulnerable to the dangers of HCIDs. This vulnerability is created partly from a lack of consistent licensing practices, education requirements related to infectious disease, and the sub-optimal application of standard precautions throughout the EMS profession. Changes are necessary to address these embedded vulnerabilities. While no one solution will effectively eliminate the current HCID vulnerability in EMS practice, a combination of approaches and a concerted effort by the EMS community can significantly improve EMS safety and reduce the risk related to HCIDs.

## Author Contributions

TR developed the concept and wrote the initial manuscript with support and guidance from CH and JO'N. CH included RF and RS to serve as subject matter experts in the field of Emergency Medicine. All authors contributed to the review process and approved the submitted version.

## Conflict of Interest

The authors declare that the research was conducted in the absence of any commercial or financial relationships that could be construed as a potential conflict of interest.

## Publisher's Note

All claims expressed in this article are solely those of the authors and do not necessarily represent those of their affiliated organizations, or those of the publisher, the editors and the reviewers. Any product that may be evaluated in this article, or claim that may be made by its manufacturer, is not guaranteed or endorsed by the publisher.
